# C and N stable isotopes enlighten the trophic behaviour of the dugong (*Dugong dugon*)

**DOI:** 10.1038/s41598-023-50578-3

**Published:** 2024-01-09

**Authors:** Martin Thibault, Yves Letourneur, Christophe Cleguer, Claire Bonneville, Marine J. Briand, Solène Derville, Paco Bustamante, Claire Garrigue

**Affiliations:** 1 Centre d’Écologie et des Sciences de la Conservation (CESCO), Muséum National d’Histoire Naturelle, Station de Biologie Marine, 1 Place de la Croix, 29900 Concarneau, France; 2 UMR ENTROPIE (UR-IRD-IFREMER-CNRS-UNC), Labex-CORAIL, 98800 Nouméa, New Caledonia; 3 Centre for Tropical Water and Aquatic Ecosystem Research (TropWATER), James Cook University, Townsville, Australia; 4 CRIOBE, USR 3278 EPHE-CNRS-UPVD, LabEx « Corail », PSL Research University, Université de Perpignan, Avenue Paul Alduy, 66860 Perpignan Cedex, France; 5 Littoral Environnement Et Sociétés (LIENSs), UMR 7266, CNRS-La Rochelle Université, 2 Rue Olympe de Gouges, 17000 La Rochelle, France; 6 Opération Cétacés, BP 12827, 98802 Nouméa, New Caledonia

**Keywords:** Behavioural ecology, Conservation biology, Ecology, Stable isotope analysis

## Abstract

The dugong (*Dugong dugon*), a large marine mammal herbivore of the Indo-Pacific, is vulnerable to extinction at a global scale due to a combination of human-related threats including habitat degradation. The species forages on seagrass habitats (marine phanerogams) and plays a key role in the functioning and sensitivity of these declining coastal ecosystems. The trophic behaviour and plasticity of dugong populations in response to extrinsic and intrinsic factors are therefore crucial features to both dugong and seagrass conservation. Yet, this knowledge remains limited to few visual observations and analyses of mouth, stomach or faecal contents of stranded individuals. We take advantage of a long-term monitoring of stranded individuals from the endangered New Caledonian population to depict features of dugongs’ trophic ecology from Carbon and Nitrogen stable isotopes. A total of 59 dugong skin samples were used to portrait the stable isotope niche of dugongs according to their sex and maturity. In light of previous work conducted in New Caledonia, a subset of these samples was used to model the trophic mix of dugong males and females. Our stable isotope mixing models used C and N isotope values of 10 taxa bbelonging to five divisions of metazoans, plants, and chromists. Our results represent the first estimate of the species dietary niche in the isotopic space. They suggest that the diet of dugong calves overlaps more with that of adult females (δ^13^C: − 6.38 ± 1.13 ‰; δ^15^N: 2.49 ± 1.10 ‰) than males (δ^13^C: − 5.92 ± 1.10 ‰; δ^15^N: 3.69 ± 1.28 ‰). Further, we highlight differences in the expected trophic mix of dugong adult males and females. From these, we formulate a sex-specific foraging behaviour hypothesis in dugongs, whereby lactating females could forage over smaller spatial ranges but more diverse food sources thanmales. The study emphasizes the importance of long-term stranding monitoring programs to study the ecology of marine mammals.. Finally, it depicts an ecological feature that may contribute to the sensitivity of vulnerable dugongs to ongoing changes on tropical coastal ecosystems.

## Introduction

The dugong (*Dugong dugon*), distributed in coastal areas of the Indo-Pacific, belongs to the highly restricted category of large marine mammal herbivores^1,2^. The species is currently considered vulnerable to extinction at a global scale as a consequence of a combination of human-related threats such as entanglement in fishing gear, collisions with ships, hunting and poaching, and habitat degradation^3^. Dugongs are particularly dependent on seagrass habitats (marine phanerogams) as food resources, which are also essential to the subsistence of many human coastal communities. Globally, seagrass habitats are declining at a rate of 7%/year due to long-term or acute environmental degradation^4^. Such a decline is alarming given the great contribution of seagrass habitats to carbon sequestration, recognized as a Nature-Based Solution to climate change by the IPCC^5^. In addition, the loss of seagrass habitats could have cascading effects on many trophic levels, including on dugongs. For instance, extreme climatic events affecting the coastal waters of eastern Australia have drastically impacted seagrass communities, which in turn resulted in lower calf production^6^ and higher mortality of dugongs^7^. Trophic interactions between dugongs and marine phanerogams is therefore likely to play a key role in the functioning and sensitivity of tropical coastal ecosystems^8^.

Dugongs feed exclusively on the seabed, their deflected snout enabling them to excavate and crop seagrass beds^9^. Seagrass constitutes the most important component of their diet, with at least nine genera consumed across their distribution range^2,10–14^. Early research suggested that dugongs specifically targeted pioneer seagrass species of the *Halodule* and *Halophila* genera^15,16^, although it is now believed that the dugongs’ diet varies across locations, seasons, and seasons within locations^2^. Both quality (nutrients) and quantity (biomass) are at play in the foraging strategies of dugongs^17–20^. The selection of seagrass patches by dugongs is also driven by a range of extrinsic factors including tides and time of the day^18,21–24^, bathymetry^25,26^, predation from sharks^27–29^ as well as seasons that control the growth and development of seagrass^30,31^. Although considered seagrass community specialists’^2^ dugongs exhibit some flexibility in their dietary characteristics, as they occasionally exploit macroalgae and macro-invertebrates^15,32^. As dugongs have been shown to play an important functional role in the growth, resilience, and dispersal of seagrass habitats^4,16,19,33^, acquiring a better understanding of their diet variability in response to extrinsic and intrinsic factors is crucial to both dugong and seagrass conservation.

While satellite tracking matched with spatially concurrent seagrass *in-situ* surveys have provided valuable insights into the foraging behaviour of dugongs^18,22,34,35^, knowledge of their diet remains limited to few visual observations (e.g., feeding herds^33^; grazing tracks^20,34^) and analyses of mouth, stomach or faecal contents of stranded individuals^11,36,37^. However, these investigations are either geographically limited to a few study sites (grazing tracks) or to few (likely unhealthy) individuals whose diet is assessed over a very short time window (gut content from stranded individuals). Skin tissues of sirenians have average turnover rates of about 50 days^38^ and integrate molecular traces of food intakes over this timeframe. An opportunity therefore exists in the study of carbon (δ^13^C) and nitrogen (δ^15^N) stable isotopes to infer spatiotemporal variations in the feeding ecology of dugongs^39^. The δ^13^C is considered an indicator of the primary production at the base of the food-web, and varies with feeding habitats^40^. The δ^15^N provides information on food/consumer relationships, as its value increases consistently with the consumer's position in the trophic network^41^. Both isotopes are widely used to study trophic features of the ecology of marine mammals^42^.

New Caledonia is located in the southwestern Pacific Ocean, at the eastern limit of the dugong range and hosts a population that has just been reclassified as endangered by the IUCN^43^. It is restricted to a few hundred individuals^44^ and suffers a low level of genetic diversity^45^, which makes it intrinsically vulnerable to both environmental and anthropogenic pressures. Ongoing research efforts on dugongs’ movements ecology outline local variations in habitat use within the complex coral reef lagoons that surround New Caledonia main island^24,35^. Yet, effective conservation planning lacks a description of dugongs’ trophic ecology at the local scale to enlighten their functional role in reef ecosystems as well as their sensitivity to habitats degradation, anthropogenic activities, and climate change.

**Figure 1:**
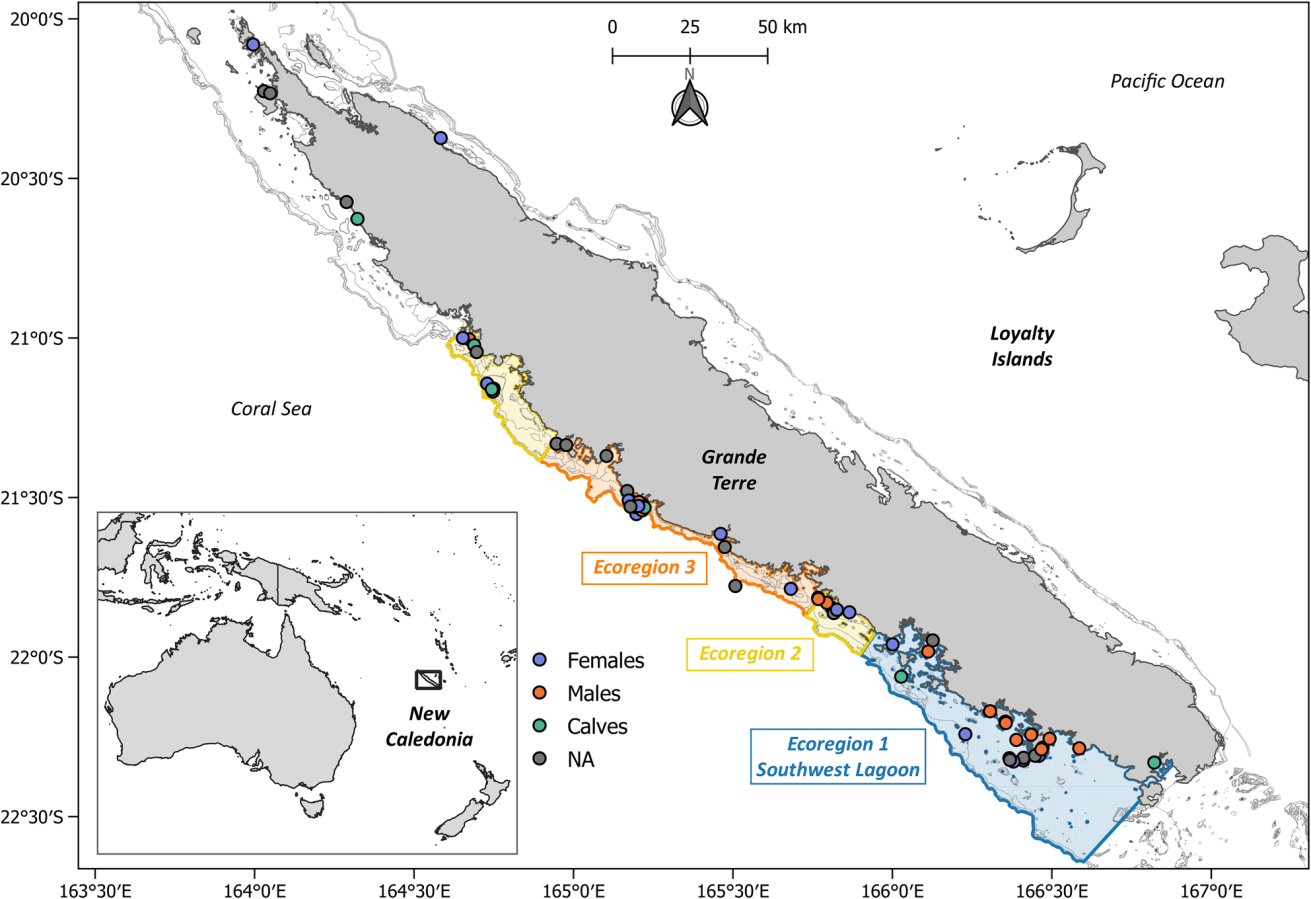
Study area and sampling distribution. Location of New Caledonia in the Southwest Pacific Ocean and the 59 individuals from which skin samples were collected. The orange dots represent adult male dugongs, adult females are represented as purple dots and calves as green dots. Ecoregions are mapped as adapted from Derville et al.^24^. Created with Qgis v3.24.3 (https://qgis.org/fr/site/).

The present study aims at providing an initial assessment of the trophic nicheand behaviour for the New Caledonia dugong population (Fig. 1). As opportunities to directly study the diet of living wild individuals are scarce, it was inferred from skin C and N stable isotope values of both tagged and stranded individuals. This opportunistic sample collection allowed to (1) describe the trophic niche of the local dugong population, (2) explore variations in the dugongs’ niche in relation to sex and age class and (3) estimate the dominant trophic mix of dugongs. From the available knowledge on dugongs’ ecology in its Pacific range, we expected isotope proxies to reflect the species herbivory through a preferential feeding on marine phanerogams. We also hypothesised that the trophic niches of adult females and calves may overlap as a result of the long parental care provided by female dugongs.

## Results

### Isotopic niche analyses

**Figure 2:**
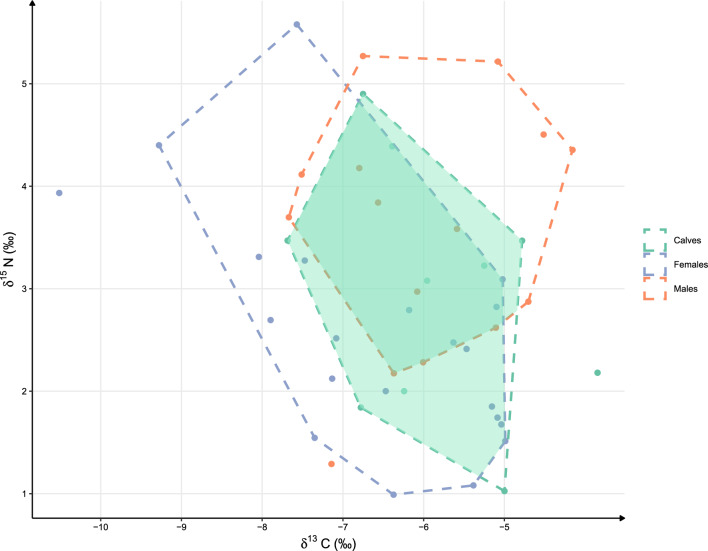
Dugongs’ trophic niches. Biplots of δ^15^N and δ^13^C of dugong skin samples. Coloured dashed lines represent the three different groups of individuals considered (calves: green; females: blue; males: orange). Dotted lines represent the α-MCP for each group. Overlaps between the α-MCP of calves and those of adult males and females are represented in plain green.

The isotopic niche of adult females (n = 21, Supplementary Table S1) was wider than that of adult males (n = 15) and calves (n = 10), irrespective of the metric used (i.e. TA, TA_95_, Table 1). The TA_95_ of dugong calves, adult males, and adult females were respectively 6.71, 7.45, and 8.85 (Fig. 2).

**Table 1:** Summary for dugong calves, adult males, and adult females of the αMCP-isotopic niches (TA, TA_α_), as well as barycentric coordinates, overlap ratios and *p* values of Hotelling niche segregation tests.

Metric	Calves	Adult males	Adult females
TA	7.99	9.00	13.68
TA_95_	6.71	7.45	8.85
Barycentre δ^13^C	− 6.09 ± 0.95 ‰	− 5.92 ± 1.10 ‰	− 6.38 ± 1.13 ‰
Barycentre δ^15^N	3.04 ± 1.24 ‰	3.69 ± 1.28 ‰	2.49 ± 1.10 ‰
Adults overlap		26.45% (*p* = 0.01)	
Intersect/calves		65.18% (*p* = 0.50)	85.57% (*p* = 0.10)

In addition to having different area sizes, the isotopic niche of male and female adult dugongs occupied statistically different positions and had a limited overlap (26.45% between the two estimated α-MCP) in the isotope space (Table 1). The significant niche segregation tests shows that the total point clouds of adult males and females were significantly different (T^2^ = 11.36, *p* = 0.009). This difference was most likely due to an overall significantly higher δ^15^N value (+ 1.2 ± 0.36, *p* = 0.002) of skin tissues in adult male dugongs (3.69 ± 1.28 ‰) compared to females (2.49 ± 1.10 ‰).

The isotopic niche of calves overlapped predominantly with that of adult females and to a lesser extent with males (Fig. 2, Table 1, respectively T^2^ = 5.22, *p* = 0.09 and T^2^ = 1.50, *p* = 0.50). More than 85% of the niche of calves was included in that of adult females compared to 65% for adult males (Table 1).

### Diet mix modelling

**Figure 3:**
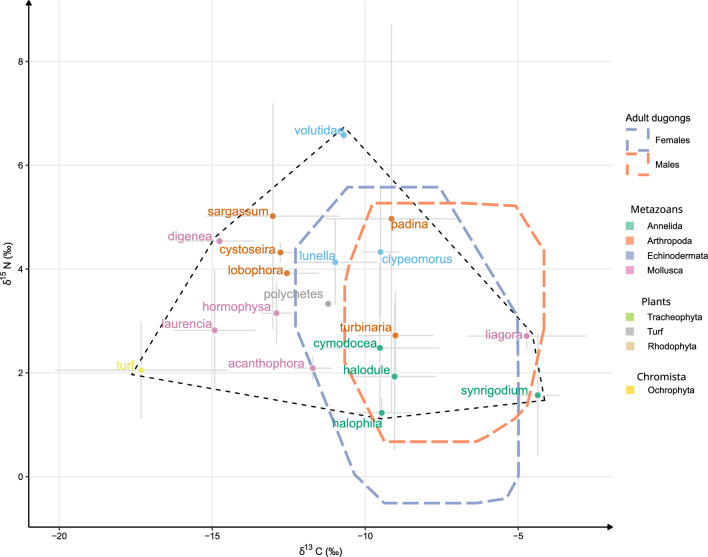
Dugongs’ niches in the resources space. Biplots of δ^15^N and δ^13^C representing the 95% MCP of adult dugong skin samples corrected with diet-skin discrimination factors (dashed lines) as well as the mean values of candidate food items ordered by taxonomic divisions in the ecoregion 1. The black dotted line represents the isotope space covered by the means of candidate genera represented by different colours (Supplementary Table S2).

**Figure 4:**
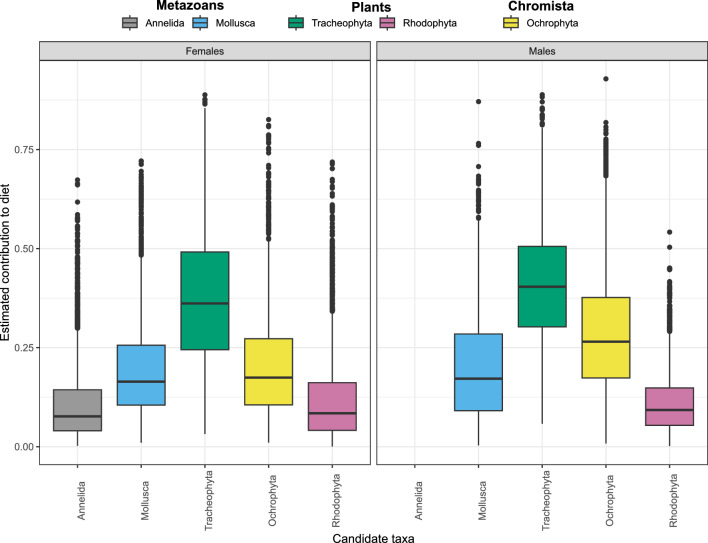
Modelled dugongs’ diet. Estimated contribution of 5 candidate food branches to the trophic mix of females (left panel) and males (right panel) dugongs in the ecoregion 1 according to candidate genera selection and the stable isotope mix modelling for both sexes (item codes in Supplementary Table S2).

Our dataset of candidate food items was made of 10 taxa belonging to five divisions of metazoans, plants, and chromists (Supplementary Table S2, Fig. 3). Modelling the relative contribution of these food items to the trophic mix of adult dugongs provided contrasted results relative to the sex of adult dugongs. Overall, female dugongs displayed a relatively more diverse diet than males (Figs. 3, 4).

The estimated 95% MCP of adult males and females extended with the discrimination factors are presented in Fig. 2. The composition of the two estimated dietary mixes of dugong males and females was mostly similar (Fig. 3, Supplementary Table S2). Marine phanerogams showed up as the most frequently consumed resource both in males (50.4%) and females (49.5%). The estimated diet of both sexes also included 20–30% of Chromista of the genera *Turbinaria* and *Padina,* and about 20% of Molluscs (*Clypeomorus* sp.*).* One Annelida genus was also included in the corrected isotope niche of dugong females and contributed to approximately 10% of the estimated trophic mix. The diet of females also integrated molluscs of the *Lunella* genus as well as *Acanthophora* red algae (Supplementary Table S2). Unfortunately, the modelled contribution of occasionally consumed items diverged fairly little from priors, particularly in females (Supplementary Fig. S1).

The four stomach contents analyzed as part of this study were dominated by plantae and chromista remains reported in Supplementary Table S3. Despite the difficulty in identifying the species due to the degradation of leaves during digestion, stomach analyses confirmed the consumption of *Halophila ovalis*, *Halodule cf uninervis*, *Syringodium isoetifolium*, *Cymodocea serrulata* and *C.* sp. Small fragments of seaweeds belonging to both genders *Sargassum* sp. and *Caulerpa* sp., as well as some shells of *Modiolus*, fragments of sponges, and ascidians were also found in the stomach of a 3.00 m-long adult female, hence emphasising the diversity of food resources ingested by dugong.

## Discussion

Although conducted on a moderate sample size, this study provides the first population-level assessment of the trophic niche of dugongs using isotopic analysis of skin samples from multiple individuals of different age class and sex. Before this study, only one study^46^ presented another isotopic analysis of dugong tissues, based on one stranded individual in Japanese waters. Our results highlight significant differences in isotopic niches and diet mix between males and females. The δ^15^N values of male skin tissues were significantly higher than that of females and calves. Our diet mix modelling approach revealed that, in New Caledonia, female dugongs have a relatively more diverse diet than males. The importance of tracheophyte species of the genera *Cymodocea, Halophila* and *Halodule* found in our study also confirms previous diet studies based on direct observation and stomach content analysis^11^*.* In this study, we show that isotopic analysis is a method of interest to shed light on the trophic behaviour of marine megafauna.

Calves shared most of their food resources with both adult males and females as shown by the non-significant niche segregation test in all cases. This reinforces the idea that young dugongs start feeding on seagrasses soon after birth, as suggested by the stomach content of a neonatal calf collected near Townsville, Australia^11^. Nevertheless, our analysis shows that the estimated isotopic niche of dugong calves overlapped three times more with that of adult females than with males. This overlap likely reflects the long lactation period of dugong females that is believed to last at least 1.5 years^47^. As they remain in close proximity with their mother until weaning, calves likely follow the same diet selection patterns potentially transmitted through mother–offspring social learning. Such diet selection transmission has been demonstrated in other terrestrial herbivores^48^ but also in marine mammals^49^.

According to the α-MCPs, the isotopic space occupied by calves was smaller than the one of adults and it overlapped with the value of only two candidate food taxa from the southwestern lagoon of New Caledonia: the plant *Halodule uninervis* and the macroalgae *Padina australis*. The former belongs to the most frequent genera found in a set of stomach contents collected from dugongs in Queensland, Australia^11^, and the latter is a species frequently observed in shallow reefs habitats in which dugongs were observed feeding in the Darwin region, Australia^50^. Together, these results suggest a similar C and N isotopic signature between calves and mothers as a result of both direct (i.e., milk consumption) and indirect (i.e., food resource selection) mechanisms. Interestingly, the calves’ estimated niche was narrower than that of adult females. Predation risk^28^, foraging and diving abilities of calves^51^, as well as physiological requirements of nursing could contribute to diet differences between lactating females, non-lactating females, and calves.

Stomach contents of dugongs contained remains of plantae dominated by three tracheophyte species: *Halodule uninervis*, *Halophila ovalis* and *Cymodocea serrulata*. This result is consistent with previous findings in analysed stomachs from Australia, where *Halodule, Halophila,* and *Cymodocea* were identified as the most common food resources (respectively 95, 89, and 61% of occurrences^11^). We also found remains of brown algae, particularly *Sargassum* sp., as documented in Japan and Australia^46,52^.

Outputs from trophic mix modelling suggested that dugongs’ diet in the southwestern lagoon of New Caledonia is made of seagrasses at more than 50% and chromists at less than 30% of assimilated items. The majority of dense and medium density seagrass meadows grow on the west and northeast coast of the main island^53^ where the highest densities of dugongs have been observed^44,54^. In the trophic mix modelling, *Halodule and Halophila* species seem to dominate the diet. These two genera are not only found in shallow coastal waters but also on the inner sedimentary terrace close to the barrier reef where dugong have also been observed^24,54^. The modelling also revealed that metazoan species such as polychaetes and small molluscs could participate in the isotopic values of dugong tissues, a result confirmed by our stomach content analysis. Deliberate consumption of invertebrates by dugongs has been reported in some studies^31^ as a response to a seasonal rarefaction of resources in winter at high latitudes^37^ and could participate to meet the nitrogen nutritive requirement of dugongs^15^. Here, metazoans species overlapped more with the trophic niche of adult females and contributed more to their estimated mix. Yet, males had higher δ^15^N values than females. One explanation to this could be the wider trophic regime of females that potentially reduces the contribution of rare food items to the global isotope value of tissues. We cannot conclude whether dugongs are targeting animal sources to meet energy requirements or accidentally consuming species associated with specific foraging habitats, namely beach rock and intertidal rocky shores for *Clypeomorus* and *Lunella* species respectively^55,56^. In such habitat, opportunistic observations of long-term feeding by dugongs on algal covered rocky reefs have been made in the Australian Northern Territory^50^. Seasonal diet selection should be further investigated to understand how dugongs adapt to the growth cycle of their preferred seagrass meadows and possibly complement their diet with other sources such as algae, molluscs, or polychaetes when seagrass availability changes.

The trophic niche of adult females was larger than that of males due to higher variations in δ^13^C within females in comparison to males. This suggests that females exploit a larger diversity of sources of organic matter. No sex or body size differences have been detected in the diet of dugongs to date except for suckling calves^10^, but sexual segregation in foraging is not well documented in sirenians, contrary to cetaceans and pinnipeds for which it has been more largely described in^57^. In coastal marine mammals with obvious sexual dimorphism, such trophic segregation between sex may arise from sex-specific feeding abilities^58^, energy requirements^59^, or reproduction-related foraging behaviour^60^. Sexual dimorphism between male and female dugongs is so low^2^ that it is unlikely to result in trophic segregation related to feeding abilities or major energy requirements.

The long gestation and lactation periods in dugongs could affect both the activity budget and the foraging selection of pregnant and lactating females^47^. Therefore, we propose a new “sex-specific foraging range” hypothesis to explain trophic assessments indicating a wider trophic niche and more resources’ contributions for females, as well as a more specialised trophic mix in males. Within these ranges, females would rely more on opportunistic and generalist feeding, contrary to males that could range further and selectively feed on a subset of the most abundant. Particularly, pregnant and lactating females could be minimising their energy expenditure by limiting their long-range movements and focusing their foraging efforts over smaller areas that may encompass a diversity of more or less optimal food resources. This hypothesis remains to test as lactating females are excluded from satellite tracking studies to prevent any risks of the tags to interfere with their movement and energy expenditure^24,35^. Linear range of movements did not significantly differ between males and females in New Caledonia^35^, but males were found to have higher residence times in specific areas when analysing space use at a 1 km scale^24^. Although no lactating females were included in these studies, results suggest sex-specific movements such as shown in other marine mammal species (e.g., Harbour seals *Phoca vitulina* from British Columbia, Canada^61^). In the northern elephant seal *Mirounga angustirostris*, such a sex-specific foraging behaviour has been identified as a result of a different tradeoff between exposition to predation and reproductive success^62^. It is therefore possible that the sex-dependent behaviours we observed could be explained by the relation between foraging behaviour and the fitness of dugong males and females.

Studying rare and endangered marine mammals with wide home ranges such as dugong is challenging from both logistical and methodological perspectives. Our work outlines the potential of long-term monitoring of stranded and poached animals to broaden our knowledge of important ecological features of marine mammals such as trophic behaviour. Maintaining such stranding monitoring networks has proved to be a substantial source of scientific data in other regions^63^, and we show how this source could be particularly precious in remote island territories (Garrigue et al., in press). Our results combine sampling of stranded individuals and the implementation of stomach content and isotope analyses.

Obviously, the use of isotope data on stranded individuals comes with challenges and limits inherent to the sampling-related heterogeneity of the data. The first challenge we encountered was the vast spatiotemporal extent over which individuals were sampled, as well as the variable degradation of stranded individuals. Indeed, trophic resources may vary spatially and with time and broad scale analyses could mask ecologically relevant spatial patterns. Similarly, diseased, starvation and tissues degradation may alter the stable isotope values of organic tissues^64^. Although the trophic niches presented here intentionally integrated spatial heterogeneity to provide a comprehensive assessment at the population scale, the mix modelling was limited to one ecoregion^24^ to limit this variability. The second challenge was the selection of the candidate species for the mix modelling, which represents a critical step in the definition of trophic relationships from isotopic data^65^. Here, we used a combination of both prior knowledge and data selection on isotope values to select candidate species. While the small sample size and the uncertainty around diet-tissues discrimination factors limited the robustness of our model outputs, their consistency with the literature^11,33,46,50^ adds confidence to this approach.

While being consistent with the knowledge acquired throughout the species range, our assessment fills a knowledge gap regarding the diet of the decreasing and vulnerable dugong population of New Caledonia. We show that (i) an estimate of the dietary niche could be drawn from opportunistically collected skin samples and the analysis of C and N stable isotopes, (ii) the dietary niche of dugong calves overlaps more with that of adult females as compared to males and (iii) the expected trophic mix of dugong adult males and females from C and N stable are different. Our isotopic niche analysis of dugongs therefore provided novel evidence for a sex-specific foraging behaviour in dugongs that is, to the best of knowledge, so far unreported in the literature. In light of the species reproductive cycle, we hypothesise that sex-specific foraging behaviour may occur in dugongs, whereby lactating females could forage over smaller spatial ranges but more diverse food sources than other individuals. More generally, this study provides important baseline knowledge of the diet of an endangered population of dugongs in a context of global seagrass habitat degradation and climate change^4^. As dugong habitat suitability and seagrass availability is likely to be altered by temperature increase, sea-level rise, extreme rainfall events, and harmful algal blooms^66^, it is crucial to estimate the current foraging behaviour of dugongs in order to predict their response to ongoing global changes.

## Methods

### Study area and samples collection

We used samples collected on dugongs along the western coast of the main island of New Caledonia (“Grande Terre”), where most dugongs are found^54,67^. A total of 59 skin samples were collected from either live (n = 25) or dead dugongs (n = 34), between 2003 and 2020 (Supplementary Table S1). Live individuals were sampled while temporarily immobilised for telemetry tag deployments^35,54,68^. Stranded (*n* = 31) and poached individuals (*n* = 3) were also sampled (Garrigue et al. in press). Handling of live animals was reviewed and approved by New Caledonia’s North Province Department of Economic Development and Environment (Permit n°609011-52), as well as South Province Department of Environment (Permits 3616-2011/ARR/DENV and 3157-2012/ARR/DENV) both in charge of reviewing the ethics of animal handlings project within their territory. Animals handling were carried out in respect relevant guidelines and regulations. Detailed methods were presented in accordance with ARRIVE in previously released movement studies^24,35,54,68^. When possible, the sex and length of sampled dugongs were determined in the field. We considered three ecoregions in the western lagoon of New Caledonia for further grouping of dugong and food item samples, according to a recent assessment of habitat use by dugongs (Fig. 4)^24^.

For the analysis, individuals smaller than two metres were considered as calves^69^. After collection, skin samples were stored in 75% ethanol until analysis. Samples were analysed following published protocols from previous works^70,71^. Subsamples of stomach contents were collected from four stranded dugongs and stored in ethanol until analysis. These contents were observed under binocular and microscope when needed to identify the organisms consumed by the dugongs whenever possible. Dominant food items were noted.

### Stable isotope analyses

Frozen skin samples from dugongs were lyophilized during 48 h before grinding. A delipidation step was then performed to avoid bias in the estimation of their δ^13^C values^72^. To that end, a few milligrams of each sample were placed in 15 ml glass tubes with 4 ml cyclohexane, agitated for 10 min and centrifuged at 4000G for 5 min and the solvent was discarded. This process was reproduced at least three times until the solvent was clear^73^. For each skin sample, ~ 0.3 mg of lipid-free powder was finally scaled and encapsulated in tin cups. Calcareous macrophytes and metazoans were divided into two sub-samples each. One sub-sample was allocated to the carbon isotope analysis; it was acidified with 1% HCl solution to remove carbonates, rinsed with distilled water and oven-dried at 40 °C for 24 h. This protocol is in agreement with the carbonates’ higher δ^13^C in comparison to organic carbon^72^. The other sample was allocated to the nitrogen isotope analysis; it was not acidified to prevent undesirable enrichment in ^15^N^74^. Samples from seagrass plus non-calcareous macrophytes and metazoans were analysed without any pre-treatment. For all cases, samples were reduced in a fine powder and ~ 0.5 mg was encapsulated in tin cups.

Isotopic analyses were conducted on a Thermo Delta V Advantage mass spectrometer coupled to a Thermo Flash EA1112 Automatic Elemental Analyzer. Results are expressed in the δ unit notation as per mil (‰) deviation from the international standards Vienna-Pee Dee Belemnite (δ^13^C) and atmospheric N_2_ (δ^15^N). Samples isotope values are reported as δ^13^C or δ^15^N values as in (1).

1 $$ \delta R_{sample} = \left[ {\left( {R_{sample} /R_{standard} } \right) - 1} \right] \times 10^{3} $$

where R is ^13^C/^12^C or ^15^N/^14^N, respectively. The analytical precision was ± 0.10 ‰ for δ^13^C and ± 0.15 ‰ for δ^15^N based on internal standards USGS-61 and USGS-62 inserted every ten measurements.

### Isotopic niche analyses

The concept of isotopic niche is, to some degree, related to the trophic niche concept and allows to calculate, within a δ^13^C–δ^15^N space, what can be considered a “trophic surface”^75^. Isotopic niches of adult females and males as well as calves were described following the method presented in^76^. Minimum Convex Polygons (MCP) were drawn on isotope biplots for the three categories of individuals and described with two metrics related to MCP surfaces (Total Area TA and Total Area 95% TA_95_). Considering our small sample sizes, we chose the most conservative threshold *α* of 95%^77^. Pairwise niche comparisons were then conducted in two steps.

The first step aimed at calculating the overlap between *α*-MCP pairs, which was calculated differently depending on the hypothesis. The overlap between isotope niches of adult males and females was calculated as the ratio between the overlapping area and the residual areas that do not overlap. This approach provides a metric that is independent from the TA*α* of males and females dugong^77^. The isotopic niche overlap between calves and adult dugongs was calculated as the ratio between the calves-adults overlapping area and the TA*α* of calves to provide a metric relative to the isotopic niche of calves.

The second step consisted in evaluating the segregation between the isotope niche of dugong calves, adult females, and adult males. This was achieved by running Hotelling T^2^ tests between pairs of isotope data subsets^78^. To lower the risk of bias associated with removing 1-*α* outliers from a heterogeneous dataset^77^, Hotelling tests were run on all available isotope data for each group. Tests were conducted with the R package “Hotelling” v1.0-8^79^.

### Resource mix modelling

Modelling of the potential trophic mix of adult dugongs was conducted in four steps following^80^. First, a list of potential food resources and their isotopic values was compiled. To lower the risk of confounding spatial effect we focused on the ecoregion 1 proposed by^24^, for which our dugong skin samples could be matched with locally collected food resource samples^70^. Second, we identified potential dugong food resources from the literature^2,15,50,81^ and averaged isotope values at the genera level to lower the number of candidate species of similar contribution in further mix models. Third, we searched for reliable diet-tissue isotope discrimination factors in the literature and found references of diet-skin values in manatees (*Trichechus manatus manatu*s and *T. m. latirostris*), cousins’ species, for both δ^13^C and δ^15^N^82^. To reduce the number of candidate species to be included in mixing models, candidate genera were filtered with a mask made of the original 95% MCP of dugong males and females (*i.e.* no discrimination applied) to which we added a 3.0 ‰ correction buffer toward lower values of δ^13^C axis and a 1.5 ‰ correction buffer toward lower values of δ^15^N. The two values were chosen as the extreme values obtained by^82^ from their dedicated assessment in manatees, the closest known assessment to our dugongs. Finally, we ran Stable Isotope Mixing Models with the package “simmr” v 0.4.5^83^. Mixing models focused on 8 adult males and 5 adult females sampled in the ecoregion 1 and considered the most realistic potential candidate genera of food resources. Candidate resources were further recombined at the branch taxonomic level to produce the final trophic mix estimates (Supplementary Table S2). Prior and posterior distributions for the two stable isotope mixing models are provided in Supplementary Fig. S1 along with and their comparison with Hellinger distances and Kolgomorov–Smirnov tests.

### Ethics

All permits required to sample carcasses of dugongs or get biopsy on living animals were obtained from New Caledonian authorities. All permits required to capture and satellite track dugongs were obtained from the James Cook University Animal Ethics Committee (Permits A1735 and A1936), Murdoch University (R3169/19), and the North (60912155-2013/JJC and N°609011-93/2018/DEPART/JJC) and South (3157- 2012/ARR/DENV) Provinces of New Caledonia.

### Supplementary Information

Supplementary Information.

## Data Availability

All the data are provided as supplementary material.
