# Supplementary material for: C and N stable isotopes enlighten the trophic behaviour of the dugong (Dugong dugon)
Source: Sci Rep. 2024 Jan 9;14:896. doi: 10.1038/s41598-023-50578-3 (PMC10776846; doi:10.1038/s41598-023-50578-3)

**Supplementary Material**

**Supplementary Table S1:** List and characteristics of the dugong individuals from which skin samples were used in this study. Provided is the origin of the samples (biopsy, stranding or poaching), the ecoregion of origin, length of the animal, and sex when available from visual determination, the maturity category to which it was included for the present study and its inclusion in either the isotopic niche analysis or the diet modelling. na: not available.

| **Origin** | **Individuals** | **skin id** | **ecoregion** | **Sex** | **Length (m)** | **δ^13^C (‰)** | **δ^15^N (‰)** | **Maturity** | **Niche Analysis** | **Diet Model** |
| --- | --- | --- | --- | --- | --- | --- | --- | --- | --- | --- |
| stranding | *ec2004-04* | *nc04-53* | 3 | M | 2.50 | -4.51 | 4.50 | Adult | yes |  |
|  | *ec2006-01* | *nc06-02* | 1 | M | 1.82 | -6.75 | 4.90 | Calf | yes |  |
|  | *ec2006-13* | *nc06-170* | 1 | M | 2.54 | -6.75 | 5.27 | Adult | yes | yes |
|  | *ec2007-06* | *nc07-161* | 1 | M | 2.60 | -6.80 | 4.18 | Adult | yes | yes |
|  | *ec2008-03* | *nc08-169* | 2 | *na* | *na* | -7.04 | 3.89 | *na* |  |  |
|  | *ec2009-01* | *nc09-10* | 1 | F | 2.83 | -8.04 | 3.31 | Adult | yes | yes |
|  | *ec2010-01* | *nc10-002* | 2 | M | *na* | -4.64 | 2.88 | *na* |  |  |
|  | *ec2010-02* | *nc10-009* | 2 | *na* | *na* | -3.11 | 5.19 | *na* |  |  |
|  | *ec2011-02* | *nc11-017* | 2 | *na* | *na* | -5.83 | 3.64 | *na* |  |  |
|  | *ec2011-05* | *nc11-265* | 3 | M | 1.16 | -5.25 | 3.22 | Calf | yes |  |
|  | *ec2012-01* | *nc12-001* | 1 | F | 2.60 | -4.99 | 1.51 | Adult | yes | yes |
|  | *ec2012-02* | *nc12-158* | 3 | F | 2.10 | -6.18 | 2.79 | Adult | yes |  |
|  | *ec2012-03* | *nc12-159* | 1 | *na* | *na* | -4.68 | 3.28 | *na* |  |  |
|  | *ec2012-04* | *nc12-155* | 1 | M | 2.56 | -7.51 | 4.11 | Adult | yes | yes |
|  | *ec2012-05* | *nc12-156* | 1 | M | 2.53 | -4.16 | 4.35 | Adult | yes | yes |
|  | *ec2014-01* | *nc14-001* | 1 | M | 2.55 | -5.59 | 3.58 | Adult | yes | yes |
|  | *ec2014-03* | *nc14-077* | 2 | *na* | 1.90 | -3.85 | 2.18 | Calf | yes |  |
|  | *ec2014-04* | *nc14-079* | 2 | F | 2.30 | -6.37 | 0.99 | Adult | yes |  |
|  | *ec2014-06* | *nc14-078* | 3 | M | 1.70 | -7.69 | 3.47 | Calf | yes |  |
|  | *ec2015-07* | *nc15-172* | 1 | *na* | 1.20 | -6.24 | 2.00 | Calf | yes |  |
|  | *ec2015-08* | *nc15-171* | 1 | F | 2.40 | -9.28 | 4.40 | Adult | yes | yes |
|  | *ec2017-01* | *nc17-001* | *na* | F | 2.75 | -7.47 | 3.27 | Adult | yes |  |
|  | *ec2018-01* | *nc18-001* | 1 | *na* | 2.50 | -7.26 | 6.26 | Adult |  |  |
|  | *ec2019-01* | *nc19-001* | *2* | M | 3.10 | -5.11 | 2.62 | Adult | yes |  |
|  | *ec2019-02* | *nc19-002* | 1 | M | 1.25 | -5.96 | 3.08 | Calf | yes |  |
|  | *ec2019-03* | *nc19-003* | 2 | F | 2.98 | -7.90 | 2.69 | Adult | yes |  |
|  | *ec2020-01* | *nc20-001* | 3 | *na* | 2.89 | -5.66 | 1.79 | Adult |  |  |
|  | *ec2020-012* | *nc20-013* | 2 | F | 2.65 | -7.35 | 1.54 | Adult | yes |  |
|  | *ec2020-013* | *nc20-014* | 1 | M | 2.40 | -6.08 | 2.97 | Adult | yes | yes |
|  | *ec2020-02* | *nc20-002* | 1 | M | 2.10 | -5.08 | 5.22 | Adult | yes | yes |
|  | *ec2021-01* | *nc21-002* | *2* | F | 2.70 | -7.57 | 5.58 | Adult | yes |  |
| Biopsy | *162403* | *nc19-203* | 3 | F | 2.40 | -5.38 | 1.08 | Adult | yes |  |
|  | *183558* | *nc19-201* | 3 | F | 2.23 | -5.04 | 1.68 | Adult | yes |  |
|  | *183559* | *nc19-200* | 3 | F | 2.36 | -10.51 | 3.93 | Adult | yes |  |
|  | *183560* | *nc19-204* | 3 | F | 1.80 | -5.00 | 1.03 | Calf | yes |  |
|  | *183561* | *nc19-202* | 3 | F | 2.95 | -5.09 | 1.74 | Adult | yes |  |
|  | *638703* | *nc13-136* | 2 | F | 2.90 | -5.15 | 1.85 | Adult | yes |  |
|  | *638706* | *nc12-161* | 2 | F | 2.30 | -7.08 | 2.52 | Adult | yes |  |
|  | *668675* | *nc13-139* | 2 | M | 2.40 | -6.01 | 2.28 | Adult | yes |  |
|  | *668680* | *nc13-134* | 2 | F | 2.70 | -5.02 | 3.09 | Adult | yes |  |
|  | *668681* | *nc13-129* | 1 | M | 2.50 | -6.56 | 3.84 | Adult | yes | yes |
|  | *668682* | *nc13-130* | 1 | F | 2.80 | -6.47 | 2.00 | Adult | yes | yes |
|  | *668683* | *nc13-132* | 1 | F | 2.60 | -7.13 | 2.12 | Adult | yes | yes |
|  | *668684* | *nc13-138* | 2 | M | 2.20 | -7.14 | 1.29 | Adult | yes |  |
|  | *668685* | *nc13-141* | 2 | M | 2.30 | -6.37 | 2.17 | Adult | yes |  |
|  | *668686* | *nc13-137* | 2 | F | 2.30 | -5.47 | 2.41 | Adult | yes |  |
|  | *668687* | *nc13-140* | 2 | F | 2.70 | -5.10 | 2.82 | Adult | yes |  |
|  | *638703a* | *nc12-160* | 2 | M | 2.70 | -7.67 | 3.70 | Adult | yes |  |
|  | *nc03-02* | *nc03-02* | 1 | *na* | *na* | -6.29 | 3.33 | *na* |  |  |
|  | *nc03-03* | *nc03-03* | 1 | *na* | *na* | -5.14 | 3.88 | *na* |  |  |
|  | *nc13-131* | *nc13-131* | 1 | *na* | 1.95 | -6.78 | 1.84 | Calf | yes |  |
|  | *nc13-135* | *nc13-135* | 2 | F | *na* | -5.19 | 2.83 | *na* |  |  |
|  | *nc13-142* | *nc13-142* | 2 | M | 2.04 | -4.70 | 2.87 | Adult | yes |  |
|  | *nc13-143* | *nc13-143* | 2 | M | 1.95 | -4.78 | 3.47 | Calf | yes |  |
|  | *nc13-144* | *nc13-144* | 2 | F | 2.50 | -5.63 | 2.48 | Adult | yes |  |
|  | *nc21-001* | *nc21-001* | *na* | F | 1.05 | -6.39 | 4.39 | Calf | yes |  |
| Poaching | *ec2012-08* | *nc12-002* | 3 | *na* | *na* | -3.81 | 2.49 | *na* |  |  |
|  | *ec2013-09* | *nc13-163* | 3 | *na* | *na* | -6.36 | 2.64 | *na* |  |  |
|  | *ec2013-10* | *nc13-164* | 3 | *na* | *na* | -6.25 | 3.65 | *na* |  |  |

**Supplementary Table S2:** List of the candidate food taxa considered in the mixing models, and estimated contributions to the trophic mix of dugong males and females. C and N stable isotopes values (Mean ± SD, in ‰) are issued from Briand et al., 2015 and Fey et al., 2019. Genera that were used to model the trophic mix of dugong males and females respectively are marked with an x.

| **Kingdom** | **Division** | **Genera** | **δ^13^C ± *sd*** | **δ^15^N ± *sd*** | **Males mix** | | **Females mix** |
| --- | --- | --- | --- | --- | --- | --- | --- |
| ANIMALIA | |  |  |  |  |  | |
|  | Annelida |  |  |  |  | 10.8 ± 9.8 % | |
|  |  | *polychaeta* | -11.21 ± 0.13 | 3.33 ± 0.08 |  | x | |
|  |  |  |  |  |  |  | |
|  | Mollusca |  |  |  | 19.7 ± 13.2 % | 19.4 ± 12.1 % | |
|  |  | *Clypeomorus* | -9.50 ± 0.61 | 4.33 ± 1.24 | x | x | |
|  |  | *Lunella* | -10.98 ± 1.38 | 4.13 ± 0.82 |  | x | |
| CHROMISTA | |  |  |  |  |  | |
|  | Ochrophyta |  |  |  | 28.6 ± 15.0 % | 20.4 ± 13.1 % | |
|  |  | *Padina* | -9.14 ± 2.14 | 4.97 ± 3.74 | x | x | |
|  |  | *Turbinaria* | -9.01 ± 1.24 | 2.72 ± 0.86 | x | x | |
| PLANTAE | |  |  |  |  |  | |
|  | Rhodophyta |  |  |  | 10.9 ± 7.4 % | 11.9 ± 11.1 % | |
|  |  | *Acanthophora* | -11.71 ± 0.62 | 2.09 ± 0.22 |  | x | |
|  |  | *Liagora* | -4.72 ± 1.93 | 2.71 ± 0.25 | x |  | |
|  |  |  |  |  |  |  | |
|  | Tracheophyta |  |  |  | 40.6 ± 14.5 % | 37.6 ± 7.5 % | |
|  |  | *Cymodocea* | -10.17 ± 1.65 | 3.01 ± 1.24 | x | x | |
|  |  | *Halodule* | -9.46 ± 0.99 | 1.23 ± 0.29 | x | x | |
|  |  | *Halophila* | -9.04 ± 1.35 | 1.93 ± 1.41 | x | x | |

**Supplementary Table S3** List of stomach content samples on which macroscopic food remains analysis were conducted. For each sample, identified Plants, Chromists and Metazoan remains are reported. The plant item that was dominant in the whole sample in presented in a dedicated column. The ecoregion were the dugong originated is also provided.

|  | **Plantae** | |  |  | **Ecoregion** |
| --- | --- | --- | --- | --- | --- |
| **Sample-id** | **Dominant** | **Identified** | **Chromista** | **Metazoans** |  |
| **EC2006-01-01** | *Halophila ovalis* | *Cymodocea serrulata*  *Halodule uninervis* |  |  | 1 |
| **EC2006-13-01** | *Cymodocea serrulata* | *Halophila* cf. *ovalis* |  |  | 1 |
| **EC2021-03-01** | *Halodule uninervis* | *Cymodocea serrulata* | *Sargassum* sp.  Brown algae  *Caulerpa taxifolia*  *C. cupressoides* |  | 3 |
| **EC2022-02-01** | *Halodule uninervis* | *Halodule uninervis*  *Halophila* sp.  *Cymodocea serrulata*  *C. rotundata*  *Syringodium isoetifolium* | *Sargassum* sp. | Unidentified sponge, *Modiolus* sp.*,* unidentified acsidian | 1 |

**Supplementary Figure S1:** Difference between the prior (dashed) and posterior (solid) distributions of estimated proportion of food items in the diet of dugong females (A) and males (B). Hellinger distance (Hellinger) and Kolmogorov-Smirnov distribution comparison test values are provided.

A)


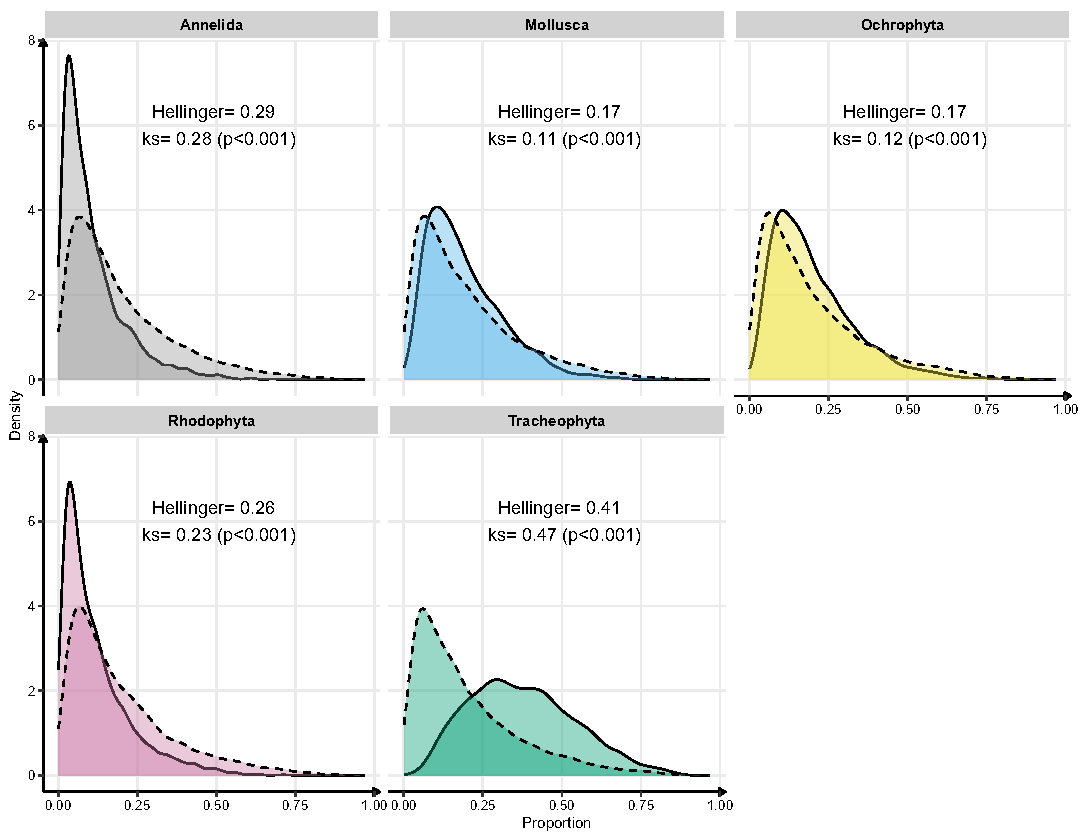


B)


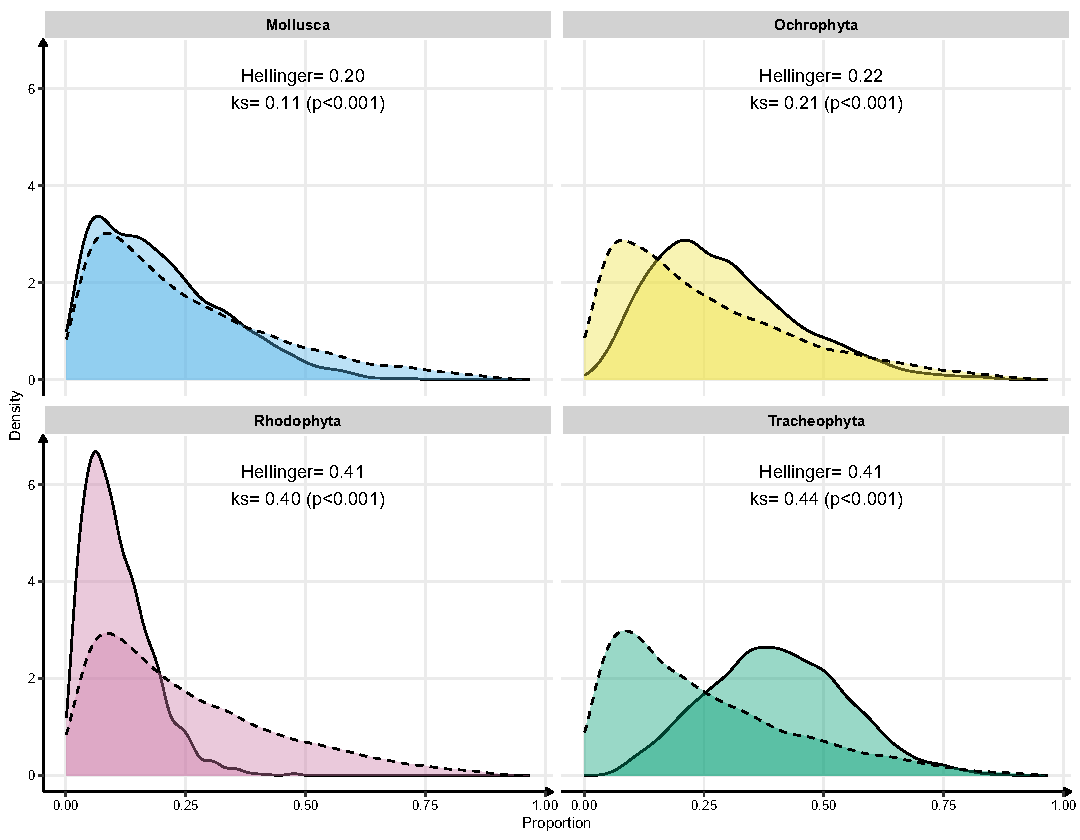

Supplement: Supplementary file 1 — Supplementary Information. [file 41598_2023_50578_MOESM1_ESM.docx]
